# Assignment of Regioirregular Sequences in the ^13^C NMR Spectrum of Syndiotactic Polypropylene

**DOI:** 10.3390/polym10080863

**Published:** 2018-08-04

**Authors:** Roberta Cipullo, Antonio Vittoria, Vincenzo Busico

**Affiliations:** Dipartimento di Scienze Chimiche, Università di Napoli Federico II, Via Cintia, 80126 Napoli, Italy; antonio.vittoria@unina.it (A.V.); busico@unina.it (V.B.)

**Keywords:** syndiotactic polypropylene, ^13^C NMR spectroscopy, regiodefects, high temperature cryoprobe, *C*_s_-simmetric *ansa*-zirconocenes, bis(phenoxyimine)Ti catalysts

## Abstract

The ^13^C NMR microstructure of a polypropylene (PP) sample is a fundamental source of information on its properties, and also a ‘fingerprint’ of the catalytic species used to produce it. Likely due to a much greater technological importance, isotactic polymers (i-PP) have been more thoroughly investigated that syndiotactic ones (s-PP). In this paper, we report the first full assignment of regioirregular sequences in s-PP samples made with two well-known molecular catalysts, namely a *C*_s_-symmetric (cyclopentadienyl)(fluorenyl) *ansa*-zirconocene and a fluxional bis(phenoxyimine)Ti species. The results shed more light on the mechanism of chain propagation at the two catalysts, and open the door to the investigation of more elusive cases like the formation of s-PP blocks in the presence of multi-sited heterogeneous Ziegler-Natta systems.

## 1. Introduction

^13^C NMR spectroscopy in solution is the technique of choice for the investigation of polyolefin microstructure [1]. In recent years, the drawback of an inherently poor sensitivity of natural abundance ^13^C NMR has been overcome by the implementation of high-temperature cryoprobes [2,3]. With an 8.5-fold enhancement of the signal-to-noise (S/N) ratio on a single pulse compared with standard probes at a given diameter, the new probes enable a 70-fold reduction of experiment time for a desired S/N value. This can be exploited in High Throughput Experimentation (HTE) applications [4,5], or aimed to the detection of rare (albeit influential) microstructural features, such as chain ends, long chain branches, or regioirregular sequences.

In the latter context, regiodefects in polypropylene (PP) are especially important. In isotactic chain propagation at classical Ziegler-Natta (ZN) and *C*_2_-symmetric *ansa*-metallocene catalytic species, which are highly regioselective in favor of 1,2 monomer insertion, it has been demonstrated that growing polymeryls with an occasional 2,1 last-inserted unit are poorly reactive towards a further propene insertion, due to the steric encumbrance of their α-methyl branch [1,6,7,8,9,10,11]. As a consequence, these ‘dormant’ chains are prone to σ-bond metathesis with molecular H_2_, and therefore are mainly responsible for the so-called ‘hydrogen response’ of a catalyst, which is key for polymer molecular weight control [1,12,13]. It has also been pointed out that the configuration of chain segments containing an isolated regiodefect in isotactic PP (i-PP) samples prepared with different catalyst classes is highly idiosyncratic, and can be combined with the classical determinations on fully regioregular sequences [1] so as to improve the effectiveness of catalyst ‘fingerprinting’.

**Chart 1:**
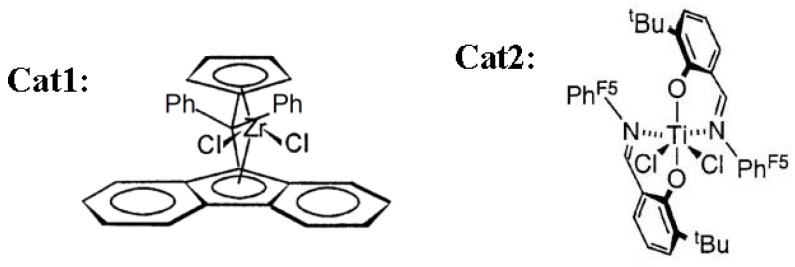
Structures of the investigated precursors.

To the best of our knowledge, the assignment of such segments in syndiotactic PP (s-PP) is still largely incomplete. Apart from a much lower technological impact of this polymer compared with i-PP, s-PP microstructure is more complex to elucidate. In the present paper, we report the first full ^13^C NMR assignment of isolated regiodefects in s-PP samples produced with two well-known molecular catalysts (Chart 1), namely the *C*_s_-symmetric stereorigid *ansa*-zirconocene **Cat1** [14,15], and the *C*_2_-symmetric fluxional bis(phenoxy-imine)Ti complex **Cat2** [16].

The results achieved in this study are not only relevant *per se,* because they clarify some fine details of the mechanism of stereocontrol of these systems in propene polymerization, but also allow the elucidation of the stereostructure of regioirregular sequences in ZN-PP, thus probing the coordination environment of the active Ti centers, as will be discussed in a dedicated paper.

## 2. Materials and Methods

**Cat1** was obtained by MCAT GmbH; **Cat2** was prepared according to the literature [16]. Propene polymerization with **CAT1** was carried out at 50 °C in a 2 L magnetically stirred (1000 rpm) stainless steel reactor (Brignole AU-2), equipped with a glass vial holder-breaker. 500 mL of dry toluene (Romil, HPLC grade, Cambridgeshire, UK) containing methylalumoxane (MAO) (Chemtura GmbH, Philadelphia, PA, USA), 10% *w/w* solution in toluene; [Al]/[Zr] = 1.0 × 10^4^), and 2,6-di-*ter*-butylphenol (Aldrich, St Louis, MO, USA); [^t^Bu_2_PhOH]/[Al] = 0.5) were charged into the reactor, which was thermostated at the polymerization temperature and saturated with propene at the partial pressure of 8.0 bar. The reaction was then started by breaking a glass vial containing the solid precursor (previously sealed under argon in a Vacuum-Atmospheres glove box), and allowed to proceed at constant propene pressure by feeding the monomer on demand. The catalyst concentration ([Zr] = 0.7 µM) was adjusted so as to keep monomer conversion below 1.0 g min^−1^, which enabled temperature control of the liquid phase within ±0.5 °C. The polymerization was stopped after 30 min by quickly venting the reactor. The polymer was coagulated with acidified methanol, filtered and vacuum dried; yield, 9.2 g. For **CAT2**, the polymerization was carried out at 20 °C and a propene partial pressure of 1.0 bar in a jacketed 250 mL Pyrex^TM^ glass reactor ([Zr] = 0.34 mM; [Al]/[Zr] = 2.0 × 10^2^), following a protocol similar to that previously described [17]; yield, 2.9 g.

Quantitative ^13^C NMR spectra were recorded using a Bruker Avance III 400 spectrometer equipped with a high-temperature cryoprobe for 5 mm OD tubes, on 45 mg mL^−1^ polymer solutions in tetrachloroethane-1,2-*d*_2_ (with BHT added as stabilizer, [BHT] = 0.4 mg mL^−1^). Acquisition conditions were: 45° pulse; acquisition time, 2.7 s; relaxation delay, 5.0 s; 2-10K transients. Broad-band proton decoupling was achieved with a modified WALTZ16 sequence (BI_WALTZ16_32 by Bruker, Switzerland).

Semiempirical calculations of the ^13^C NMR chemical shifts were made using the NCheng software [18].

## 3. Results and Discussion

### 3.1. Assignment of Isolated 2,1 Units in s-PP Made with CAT1

**Cat1** (in combination with methylalumoxane, MAO) was the first catalyst ever reported able to yield, under proper conditions (i.e., high monomer concentration and low temperature) highly stereoregular s-PP [14]. It is also one of the most regioselective zirconocene catalysts in the public domain: the favored propene insertion mode is 1,2 (primary), and the ^13^C NMR spectra of the polymer recorded with standard probes do not reveal 2,1-inserted units. Several years ago, by applying the ^13^C NMR 1-^13^C-ethene/propene copolymerization method to **Cat1**/MAO at 10 °C, we estimated a fraction of 2,1 units as low as 0.08 mol % [8,19].

**Figure 1:**
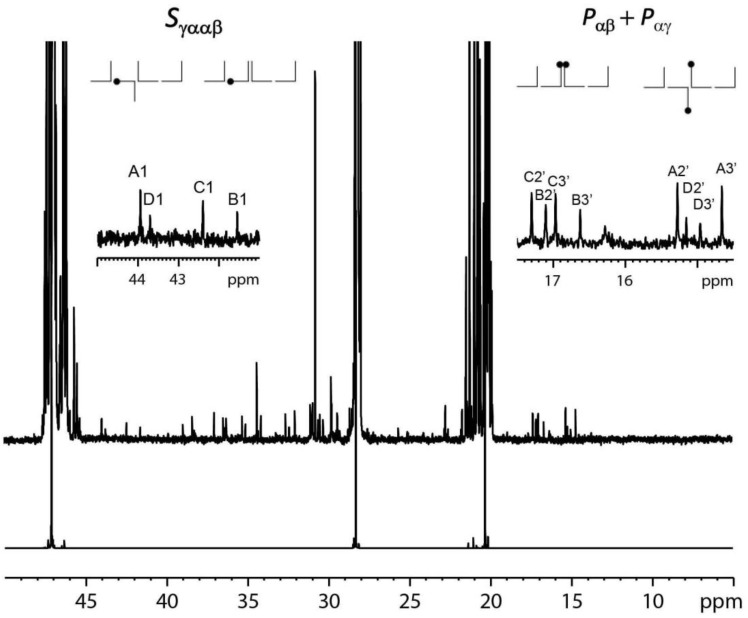
^13^C NMR spectrum of the syndiotactic polypropylene (s-PP) sample made with **Cat1**.

The quantitative ^13^C NMR spectrum of an s-PP sample prepared with **Cat1**/MAO in toluene solution at 50 °C under conditions approaching ‘kinetic quench (KQ)’ regime [20] (that is, a strict alternation of monomer insertions at the two enantiotopic active sites) is shown in Figure 1. The spectrum was collected with a 5 mm OD high-temperature cryoprobe (20 K transients). Full polymerization and polymer characterization details can be found in the Materials and Method Section.

By integration of the methyl region in the *δ* ≈ 19.8–22.0 ppm range, we measured a fraction of …*rrrrmrrrr*… stereodefects (traceable to site epimerization events [1]) of 0.8%.

Several weak resonances can be attributed to the distinctive C’s of isolated 2,1-inserted units and their 1,2-inserted first neighbors. In particular, the regions of the S_γααβ_ methylene C’s [21,22] (*δ* ≈ 41–45 ppm) and *P*_αβ_ and *P*_αγ_ methyl C’s [21,22] (*δ* ≈ 14–18 ppm) both feature four sets of resonances in a roughly 2:2:1:1 integral ratio, pointing to a corresponding number of different microstructures. Based on the methyl pattern, in two of them the head-to-head linkage is in *erythro* configuration (*δ* ≈ 14–16 ppm), whereas in the other two it is in *threo* configuration (*δ* ≈ 16–18 ppm). From the cumulative integral of each region, we estimated an overall fraction of 2,1 units [2,1] = 0.13% (which is in good agreement with the aforementioned indirect estimate based on 1-^13^C-ethene copolymerization, considering the higher polymerization temperature used in the present study). Here we propose an assignment based on experimental and computational molecular kinetic information, and semiempirical calculations of chemical shifts.

**Figure 2:**
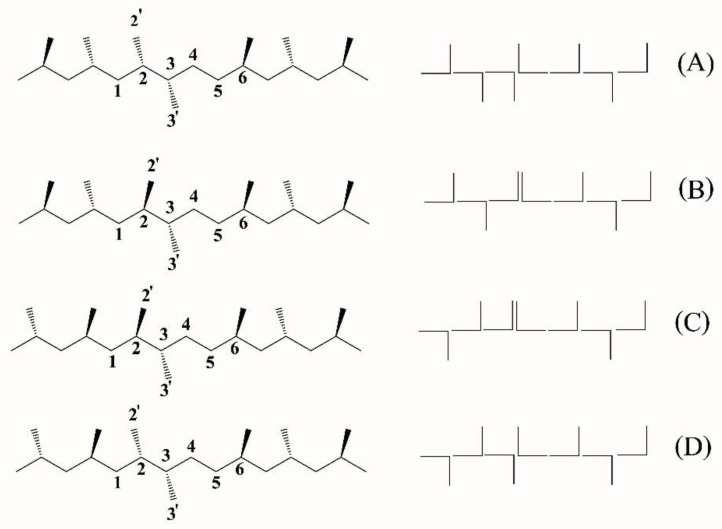
Saw-horse and Fischer projections of regioirregular sequences in the s-PP sample made with **Cat1**.

Quantum Mechanics (QM) modeling of propene insertion indicated that each active site of **Cat1** is highly enantioselective (∆∆*E*^#^ > 2.5 kcal mol^−1^) in 1,2 as well as 2,1 propene insertion in favor of the same enantioface, whereas the 1,2 insertion following a 2,1 insertion is almost nonenantioselective (∆∆*E*^#^ ≈ 0.3 kcal mol^−1^ in favor of the opposite enantioface) [23]. In view of that, under KQ regime one should expect the two microstructures labeled as **A** and **B** in Figure 2 in comparable amounts. The corresponding peaks in the spectrum of Figure 1 were attributed based on the calculated values of chemical shifts (Table 1).

The most plausible origin of the other two microstructures is an event of site epimerization that is of chain swinging between the two enantiotopic sites of **Cat1**. Under practical polymerization conditions this process is slow relative to 1,2 poly-insertion, because ion pairing with the MAO anion hampers cation symmetrization [20]. On the other hand, as was noted above, both 2,1 insertion and the 1,2 insertion following it are very slow as well (*k*_ps_ ≈ 10^-3^ *k*_pp_, *k*_sp_ < 10^−3^ *k*_pp_), which makes site epimerization more competitive. Moreover, ion pairing at a sterically encumbered active cation with an α-branched active chain-end can be expected to be weaker. Based on these arguments and the calculated values of chemical shift in Table 1, we trace the observation of microstructures **C** and **D** (Figure 2) to site epimerization after 2,1 insertion. 

### 3.2. Assignment of Isolated 2,1 Units in s-PP Made with CAT2

The second sample of s-PP was prepared with **Cat2** (Chart 1), known to produce a highly syndiotactic polypropylene with a predominantly 2,1 insertion mode [16,17,24,25]. The origin of the stereocontrol is the result of a systematic inversion of configuration of the *C*_2_-symmetric active species after each single 2,1 insertion, changing the kinetic into the thermodynamic diastereoisomer [26]. The consequence of this peculiar mechanism is a microstructure with isolated *m*-type stereodefects, mimicking a chain-end-controlled s-PP. Quantum Mechanics/Molecular Mechanics (QM/MM) calculations concluded that propene insertion into an initial Ti–Me bond is prevailingly 1,2 (∆∆*E*^#^ = 1.0 Kcal mol^−1^), but this preference vanishes for Ti–^i^Bu (∆∆*E*^#^ = 0.3 Kcal mol^−1^). More importantly, insertion into a Ti–^i^Pr bond is highly regioselective in favor of the 2,1 mode (∆∆*E*^#^ = 2.0 Kcal mol^−1^), meaning that after a 2,1 unit secondary propagation tends to be maintained [26]. As a consequence, the polymer consists of long blocks of 2,1-inserted units containing few occasional isolated 1,2 regiodefects, spanned by short blocks of 1,2 units. Head-to-head and tail-to-tail enchainments bridge the different blocks [16]. It is worthy to recall that s-PP samples with a similar microstructure were produced before with V-based ZN catalysts at low temperature [1,21,27].

**Figure 3:**
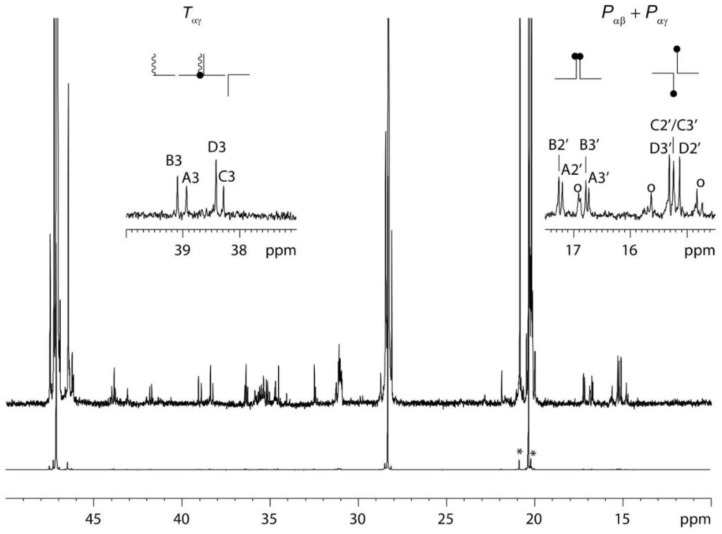
^13^C NMR spectrum of the s-PP sample made with **Cat2**. In the region of the *P*_αβ_ and *P*_αγ_ C’s, peaks marked with the (o) symbol are ascribed to 1,2 stereoblocks. The methyl peaks marked with asterisks belong to *P*_ββ_ C’s centered in *rrrmrr* and *rrrrmr* heptads.

The ^13^C NMR spectrum of the polymer made with **Cat2** is shown in Figure 3. The resonances at *δ* = 20.20 and 20.86 ppm, marked with asterisks, are due to *P*_ββ_ C’s centered in *rrrmrr* and *rrrrmr* heptads, confirming the aforementioned pseudo-chain-end microstructure [1].

**Figure 4:**
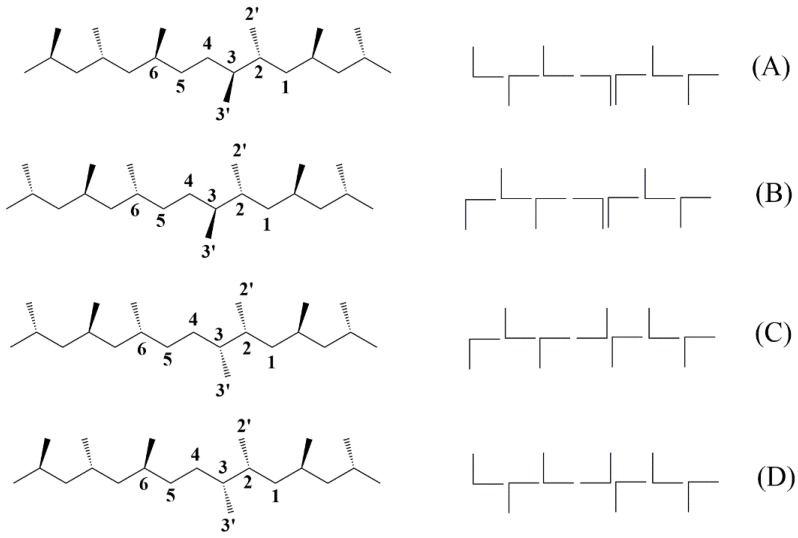
Saw-horse and Fischer projections of regioirregular sequences in the s-PP sample made with **Cat2**.

A plethora of weak peaks visible at high vertical expansion in the region between δ = 33 and 37 ppm can be attributed to *T*_αβ_ and *S*_αβ_ C’s at the stereoblock junctures. [21,28,29]. On the other hand, ‘only’ four peaks are observed in the *T*_αγ_ region between δ = 37.5 and 39.0 ppm, diagnostic for isolated 1,2 units inside syndiotactic 2,1 stereoblocks [29]. Two such peaks, at 38.5 and 39.0 ppm, are in common with the ^13^C NMR spectrum of Figure 1, and can be attributed to microstructures **B** and **D** of Figure 4, *locally* identical to **B** and **D** of Figure 2 even though they originate from opposite regioselectivities; this assignment is confirmed by the observation of all other peaks in the two calculated chemical shift sets (Table 1). In the case of **Cat2**, in our opinion, the most plausible explanation for the aforementioned structures is that, under syndiotactic 2,1 propagation, an occasional 1,2 regioerror and a subsequent 2,1 insertion are both stereoirregular; this suggests, in particular, that the steric demand of a β-branched active chain-end is not high enough to drive the ∆/Λ interconversion of the fluxional catalytic species to one configuration. If our interpretation is correct, then microstructures **A** and **C** of Figure 4 should also be observed in roughly equal amounts. Based on semiempirical calculations of chemical shifts [18], this seems indeed to be the case (Table 2 and Figure 4).

The resonances of C atoms 2 and 6 of structures **A** and **C** in Figure 4 overlap with those of consecutive 1,2 units, and at this stage their assignment is still ambiguous. From the integral values of the *T_αγ_* and *P*_αβ_ + *P_αγ_* resonances, we estimated that approximately 75% of the 1,2 units are isolated, while roughly 25% are in sequences of two or more.

## 4. Conclusions

In this paper, we have reported the first full assignment of the isolated regioirregular units in the ^13^C NMR spectra of s-PP samples obtained with a *C*_s_-symmetric *ansa*-zirconocene (**Cat1**) and a *C*_2_-symmetric bis(phenoxiimine)Ti-based (**Cat2**) propene polymerization catalyst. The elucidation of the stereochemical environment of such units shed light on the mechanism of stereocontrol for the two catalysts. In particular, in the case of **Cat1** evidence was provided that site epimerization is much more likely to occur after a regioiregular 2,1 than after a regioregular 1,2 insertion. For **Cat2**, in turn, it was found that β-branched active chain-ends are not able to direct the fluxional ∆/Λ interconversion of the catalytic species to one or the other configuration; this provides an explanation why 2,1 propagation at this catalyst is syndiotactic-selective, whereas 1,2 propagation is substantially not enantioselective.

We anticipate that the new results will also be key to interpret the microstructural fingerprint of the syndiotactic-selective catalytic species in heterogeneous Ti-based ZN systems.

## Figures and Tables

**Table 1:** Experimental and calculated [18] chemical shift values of the microstructures in Figure 2.

	1	2	2’	3	3’	4	5	6
A_Calc._	44.13	35.11	15.76	37.68	14.85	32.5	36.1	31.26
A_Exp._	44.1	34.3	15.4	37.2	14.8	32.7	36.4	31.1 ^(a)^
B_Calc._	42.32	35.83	17.1	38.53	16.76	30.77	36.70	31.30
B_Exp._	41.7	35.2	17.3	39.1	16.8	30.5	36.5	31.1 ^(a)^
C_Calc._	42.33	35.84	17.63	38.53	17.14	30.46	36.70	31.30
C_Exp._	42.6	35.4	17.5	38.5	17.1	30.6	36.6	31.2
D_Calc._	44.12	35.10	15.25	38.08	15.34	32.32	36.12	31.25
D_Exp._	43.9	34.6	15.1	38.4	15.3	32.5	36.4	31.1 ^(a)^

^(a)^ Tentative assignments.

**Table 2:** Experimental and calculated [18] chemical shift values of the microstructures in Figure 4.

	1	2’	3	3’	4	5
A_calc._	42.22	17.03	38.44	16.66	30.71	36.68
A_exp._	41.9	17.2	38.9	16.7	30.9	36.4
C_calc._	44.16	15.33	37.99	15.24	32.26	36.20
C_exp._	43.9	15.2	38.3	15.2	32.5	36.3
